# Sub-Minimum Inhibitory Concentrations of Rhubarb Water Extracts Inhibit *Streptococcus suis* Biofilm Formation

**DOI:** 10.3389/fphar.2017.00425

**Published:** 2017-07-07

**Authors:** Wen-Ya Ding, Yan-Hua Li, He Lian, Xiao-Yu Ai, Yu-Lin Zhao, Yan-Bei Yang, Qiang Han, Xin Liu, Xue-Ying Chen, Zhonggui He

**Affiliations:** ^1^Department of Pharmacy, Shenyang Pharmaceutical UniversityShenyang, China; ^2^Department of Veterinary Medicine, Northeast Agricultural UniversityHarbin, China; ^3^School of Pharmacy, Nankai UniversityTianjin, China

**Keywords:** *Streptococcus suis*, biofilm, rhubarb water extracts, two-component signal transduction system (TCSs), transcriptional regulator, DNA binding protein

## Abstract

*Streptococcus suis* is one of the most important swine pathogens, which can cause persistent infection by forming biofilms. In this study, sub-minimum inhibitory concentration (sub-MIC) of rhubarb water extracts were found to inhibit biofilm formation. Two-component signal transduction systems (TCSs), transcriptional regulators, and DNA binding proteins were compared under two conditions: (1) cells treated with sub-MIC rhubarb water extracts and (2) untreated cells. Using an isobaric tags for relative and absolute quantitation (iTRAQ) strategy, we found that TCSs constituent proteins of histidine kinase and response regulator were significantly down-regulated. This down-regulation can affect the transfer of information during biofilm formation. The transcriptional regulators and DNA binding proteins that can interact with TCSs and interrupt gene transcription were also significantly altered. For these reasons, the levels of protein expressions varied in different parts of the treated vs. untreated cells. In summary, rhubarb water extracts might serve as potential inhibitor for the control of *S. suis* biofilm formation. The change in TCSs, transcriptional regulators, and DNA binding proteins may be important factors in *S. suis* biofilm inhibition.

## Introduction

*Streptococcus suis* (*S. suis*) is a Gram-positive bacterium and it is considered to be one of the most important swine pathogens worldwide. It can cause meningitis, arthritis, septicemia, bronchopneumonia, and other pathological conditions (Zhao et al., 2015). At present, at least 29 *S. suis* serotypes have been identified. *S. suis* serotype 2 (SS2) is considered to be the most virulent for both humans and swine. In China, up to 70% of *S. suis* isolates accounting for systemic diseases in piglets are serotype 2. The diseases caused by *S. suis* 2 are difficult to cure, as *S. suis* serotype 2 can induce persistent *in vivo* infections as a result of biofilm formation (Zhao et al., 2015).

Biofilm is a community of microorganisms adhering to each other on biotic or abiotic surfaces, and the bacteria in biofilm are surrounded by self-produced matrix of extracellular polymers that can offer advantages for the microorganisms (Khoramian et al., 2015). Biofilm can reduce the penetration of antimicrobial agents and increase protection against the host immune system. Compared with free-floating bacteria of the same species, biofilm can induce as much as 1000-fold resistance to detergents, antiseptics, and antibiotics (Rasamiravaka et al., 2015). Bacterial biofilm plays an important role in persistent infections, which are rarely eradicated by antimicrobials (Costerton et al., 1999). Thus, inhibiting biofilm formation may be an important strategy for eradicating persistent bacterial infections.

Rhubarb is a drug described in the Chinese Pharmacopoeia, and it has important clinicial applications. The active ingredients of rhubarb are anthraquinones, which have been reported to inhibit the growth of viruses, bacteria, and biofilm. These mainly comprise emodin, rhein, chrysophanol, aloe-emodin, and physcion (Lin et al., 2008; Wierzchacz et al., 2009). Rhein can impair the pathogenicity of *Porphyromonas gingivalis* pathogenicity by intervening in the transcription of genes, these genes code important virulence factors which affect the bacterial proteolytic activity of the bacterium (Liao et al., 2013). Aloe-emodin can disrupt bacterial membranes by interposing between the two major phospholipids (phosphatidylethanolamine and phosphatidylglycerol) (Liao et al., 2013) that are present in bacterial membranes. It also shows antacid activity against *S. mutans* biofilm (Kim et al., 2011). Emodin can inhibit of *Pseudomonas aeruginosa* and *Stenotrophomonas maltophilia* biofilm formation (Alves et al., 2004). These anthraquinone monomers are the principal components of the rhubarb water extracts, and their effects on *S. suis* biofilm are currently unknown; therefore, in our study, we aimed to characterize the inhibitory effects of rhubarb water extracts on the formation of *S. suis* biofilm.

Two-component signal transduction systems (TCSs) are some of the most important cell-to-cell communication systems, and they play a key role in biofilm formation (Rasamiravaka et al., 2015). They consist mainly of a membrane-bound sensor histidine kinase (HK), which perceives a stimulus, and a cytoplasmic response regulator (RR), which mediates the response to the stimulus (Nobile et al., 2012). Biofilm is a multicellular aggregate of micro-organism, and its formation has been shown to be specifically involved with recognizing and responding to small self-generated secreted molecules (Rasamiravaka et al., 2015). The TCSs can recognize small molecules via HK and transmit information through the RR via conserved phosphorylation and dephosphorylation reactions (Jung et al., 2012; Parellada et al., 2013; Siddiqui et al., 2015). The final activated RR can interact with transcriptional regulators and DNA binding proteins, thereby interrupting gene transcription (Siddiqui et al., 2015; Zhao et al., 2015).

In our study, the inhibitory effects of the rhubarb water extracts on *S. suis* biofilm formation and the mechanisms responsible for this inhibition were investigated. To gain insight into these processes, tissue culture plate (TCP) and scanning electron microscopy (SEM) assessments were used to test the inhibitory effects of rhubarb water extracts on biofilm formation, and the iTRAQ technique was applied to measure variations in TCSs, transcriptional regulators, and DNA binding proteins between untreated and rhubarb water extracts-treated cells.

## Materials and Methods

### Bacterial Cultures and Biofilm Formation

*Streptococcus suis* strain ATCC 700794 was grown at 37°C with constant shaking in Todd–Hewitt broth (THB; Sigma–Aldrich, St. Louis, MO, United States) containing (w/v) 0.5% beef extract, 0.3% yeast extract, 2% peptone, 2% calf serum, 0.5% glucose, 0.04% Na_2_HPO_4_, 0.25% Na_2_CO_3_ and 0.2% NaCl. For biofilm cultures, *S. suis* was grown in THB medium (Oxoid, Wesel, Germany) supplemented with 1% fibrinogen in 200 mm polystyrene petri dishes at 37°C for 72 h. The supernatant was then removed and the petri dishes were rinsed twice with 50 mM Tris/HC1 (pH 7.5). Biofilm was scraped, and cells were sonicated for 5 min (Bransonic 220; Branson Consolidated Ultrasonic Pvt Ltd., Australia) followed by centrifugation at 4°C for 10 min at 12,000 × *g*. The supernatant was then discarded. Cell pellets were rinsed twice with 50 mM Tris–HC1 (pH 7.5) and collected by centrifugation (14,000 rpms). *S. suis* planktonic cells were grown at 37°C for 24 h. The cells were collected and washed as described above.

### Rhubarb Water Extracts Preparation and Anthraquinones Analysis

Rhubarb (Tongren Tang Co., Ltd., Beijing, China) 200 g was smashed and soaked at room temperature in 2 L water for 12 h. The suspension was then boiled for 45 min and filtered through gauze. The previous procedures were then repeated but 1.5 L water was added, and the suspension was boiled for 30 min. The two filtrates were combined and concentrated to 100 mL, and then lyophilized.

The anthraquinones content of rhubarb water extracts was analyzed by high-performance liquid chromatography (HPLC) using an RP-C18 column. The mobile phase was methanol–0.1% phosphoric acid water (85:15, v/v) at a flow rate of 1.0 ml/min at room temperature (25°C). Twenty microliters of sample was injected and monitored at 254 nm after they were diluted by the mobile phase. All assays were performed in triplicate.

### Effect of Rhubarb Water Extracts on Biofilm Formation Determined by the TCP Assay

The minimum inhibitory concentration (MIC) of rhubarb water extracts against *S. suis* was determined by the microtiter method as described in the Clinical and Laboratory Standards Institute guidelines, but with Mueller–Hinton broth was replaced by THB.

*Streptococcus suis* ATCC 700794 in mid-exponential growth phase was adjusted to an optical density of 0.2 at 600 nm (OD_600_). One-hundred microliters of culture and 100 μL of rhubarb water extracts solution were added to each well of 96-well microplate, and the final concentrations of rhubarb water extracts were 1/2, 1/4, 1/8, or 1/16 × MIC. The cells suspension without rhubarb water extracts was added for the negative controls. After incubation for 72 h without shaking, wells were rinsed with 50 mM phosphate-buffered saline (PBS; pH 7.2), fixed for 30 min with 200 μL of methanol, stained with 200 μL of 1% crystal violet (w/v) for 30 min, rinsed three times with PBS (pH 7.2), and dried for 2 h at 37°C. Finally, 200 μL of 33% acetic acid (v/v) was added, and the plate was shaken for 10 min. All wells were measured using a Tecan GENios Plus microplate reader (Tecan, Austria) at 595 nm (Zhao et al., 2015).

Sub-MIC rhubarb water extracts were added into cell suspension at 0, 24, or 48 h, and cell suspension without rhubarb water extracts served as negative controls. After incubation for 72 h without shaking, the wells were monitored, using a Tecan GENios Plus microplate reader (Tecan, Austria) at 600 nm. All assays were performed in triplicate.

### Scanning Electron Microscopy (SEM)

The *S. suis* ATCC 700794 biofilm was tested by SEM as described by Zhao et al. (2015). Briefly, a mid-exponential *S. suis* growth culture was diluted to an optical density of 0.1 at 600 nm (OD_600_), and 1 mL cell suspension was added to 6-well microplate (Corning Co., Ltd., America) in which each well contained an 11 mm × 11 mm sterilized rough organic membrane (Mosutech Co., Ltd., Shanghai, China) on the bottom. The plates were incubated at 37°C for 72 h without shaking, the supernatant was removed, and the organic membranes were rinsed with sterile PBS. Both untreaded and 1/2 MIC rhubarb water extracts-treaded bifilm were fixed with 4% (w/v) glutaraldehyde for 6 h, then washed three times with 0.1 M PBS, and fixed to black transparency with 2% (w/v) osmium tetroxide. After dehydration and critical point drying, samples were sputtered gold-sputtered using an ion sputtering instrument (current 15 mA, 2 min) and examined by scanning electron microscopy (FEI Quanta, The Netherlands).

### Determination of the Growth Inhibition Activity of Rhubarb Water Extracts

The growth of both untreated and *S. suis* treated with 1/2 MIC of rhubarb water extracts was monitored. The samples were incubated at 37°C for 48 h, and OD_600_ measurements were taken at 4, 6, 8, 10, 12, 24, and 48 h. All assays were performed in triplicate (Wisniewski et al., 2009).

### Protein Digestion and iTRAQ Labeling

Protein digestion was carried out according to the filter-aided sample preparation (FASP) procedure. Briefly, 230 μg of protein was mixed with dithiothreitol and boiled for 5 min, and proteins were collected by ultrafiltration (Microcon units, 10 kD). The sample pellets were resuspended and incubated for 30 min in darkness with 100 μL of IAA (50 mM IAA in UA), samples were collected by centrifugation at 4°C for 15 min at 14,000 × *g*, and 5 μg of trypsin (Promega) in 40 μl of DS buffer was added overnight at 37°C. Finally, the resulting peptides were collected by filtration, and the UV light spectral density at 280 nm using an extinction coefficient of 1.1 of 0.1% solution was estimated.

For labeling, 30 μg of peptides from each sample were labeled with iTRAQ reagents that were dissolved in 70 μl of ethanol and labeled as (Sample1)-117 and (Sample2)-118. The labeled samples were combined and vacuum dried.

### Strong Cation Exchange (SCX) Chromatography Separation of iTRAQ Reagent-Labeled Peptides

The labeled samples were separated by SCX chromatography with a PolySULFOETHYL column (4.6 mm × 100 mm, 5 μm, 200 Å, PolyLC Inc., Columbia, MD, United States). The column was equilibrated with buffer A (10 mM KH_2_PO_4_, 25% (v/v) acetonitrile, pH 2.7) for 25 min, and the samples were separated at a flow rate of 1 mL/min, using a step gradient of 0–10% (v/v) buffer B (500 mM KCl, 25% (v/v) acetonitrile, 10 mM KH_2_PO_4_, pH 2.7) for 2 min; 10–20% (v/v) buffer B for 25 min; 20–45% (v/v) buffer B for 5 min; 45–100% (v/v) buffer B for 5 min; and 100% (v/v) buffer B for 8 min. The elution was monitored at 214 nm, and the collected fractions were desalted on a C18 Cartridge (Sigma).

### Liquid Chromatography (LC)–Electrospray Ionization (ESI) Tandem Mass Spectrometry (MS/MS) Analysis

Sample fractions were analyzed on a Q Exactive mass spectrometer (Thermo Finnigan) that was coupled to Easy nLC (Proxeon Biosystems, now Thermo Fisher Scientific). Ten microliters of each fractions were injected and loaded onto a C18-reversed phase column (100 mm × 75 μm, 3 μm) in buffer A (0.1% formic acid). The peptide mixture was separated at a flow rate of 250 nL/min with a linear gradient of buffer B (80% acetonitrile and 0.1% formic acid) controlled by IntelliFlow technology over 140 min. A full scan range from 300 to 1800 m/z was recorded in profile mode with a resolution of 70,000 at m/z for 120 min with resolution for higher-energy collisional dissociation (HCD) spectra set at 17,500 at m/z. Normalized collision energy was set to 30 eV and the underfill ratio was 0.1%. The instrument was run with the peptide recognition mode in the enabled position.

### Data Analysis and Statistics

The acquired peak-lists of all MS/MS spectra were found using MASCOT engine (Matrix Science, London, United Kingdom; version 2.2) inset with Proteome Discoverer 1.4 (Thermo Electron, San Jose, CA, United States) against the Uniprot_streptococcus_suis.fasta (38369 sequences, downloaded on November 4th, 2013) and the decoy database. The following parameters were set: Missed cleavage = 2; Enzyme = Trypsin; Peptide mass tolerance = 20 ppm; MS/MS tolerance = 0.1 Da; Fixed modification: Carbamidomethyl (C); iTRAQ8plex(N-term); iTRAQ8plex(K); Variable modification: Oxidation(M); False discovery rate ≤ 0.01.

The values were calculated as the mean of individual experiments in triplicate and compared with those of the control groups. Differences between two mean values were calculated by Student’s *t*-test using SPSS 11.0 statistical software.

## Results

### Quantitative Analysis of the Anthraquinones in Rhubarb Extracts

The rhubarb water extracts were analyzed by HPLC, and the mean contents of aloe-emodin, rhein, emodin, chrysophanol and physcion were 0.96 ± 0.0011 mg/g, 2.63 ± 0.0033 mg/g, 1.08 ± 0.0058 mg/g, 2.29 ± 0.0137 mg/g, and 1.82 ± 0.0083 mg/g, respectively (**Table 1**).

**Table 1:** Contents of aloe-emodin, rhein, emodin, chrysophanol, and physcion in rhubarb water extracts.

Name	Emodin	Rhein	Chrysophanol	Physcion	Aloe-emodin
Content (mg/g)	1.08 ± 0.0058	2.63 ± 0.0033	2.29 ± 0.0137	1.82 ± 0.0083	0.96 ± 0.0011

### Growth Inhibitory Activity and Biofilm Formation Inhibition Assay

**FIGURE 1:**
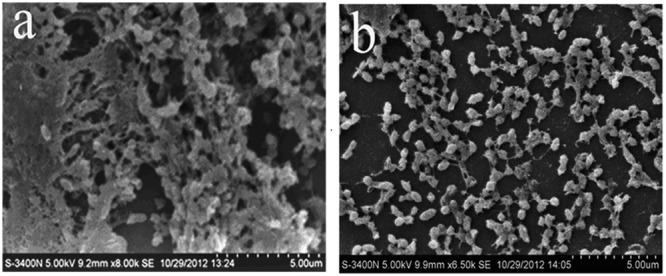
Scanning electron microscope observation. **(a)** Untreated *Streptococcus suis* ATCC700794. **(b)** *S. suis* ATCC700794 treated with 1/2 MIC rhubarb water extracts.

**FIGURE 2:**
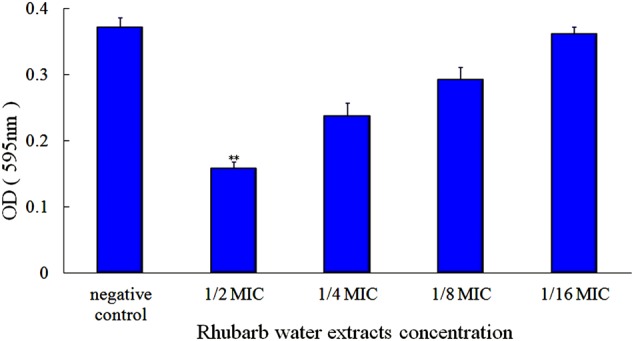
Effects of rhubarb water extracts on *S. suis* ATCC700794 biofilm formation at different concentrations. Data are expressed as means ± standard deviations (^∗∗^*p* < 0.05).

**FIGURE 3:**
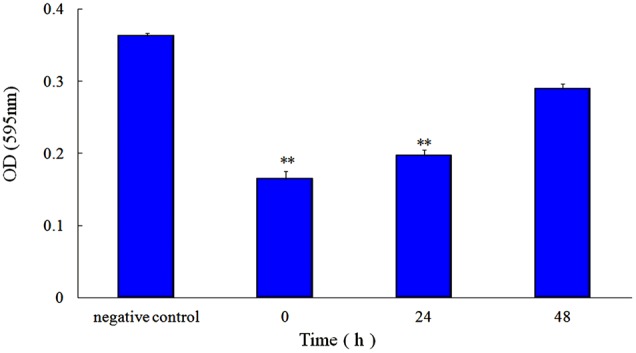
Effect of 1/2 MIC rhubarb water extracts on *S. suis* ATCC700794 biofilm formation. Data are expressed as means ± standard deviations (^∗∗^*p* < 0.05).

First, the MIC of rhubarb water extracts against *S. suis* ATCC 700794 was determined as 1.56 mg.mL^-1^. Second, sub-inhibitory concentrations of rhubarb water extracts corresponding to 1/2, 1/4, 1/8, and 1/16 MIC (0.78, 0.39, 0.20, and 0.1 mg/ml of rhubarb water extracts, respectively) were assayed by TCP to assess the inhibitory effects of biofilm formation. The results demonstrated that the rhubarb water extracts could obviously reduce biofilm formation, especially 1/2 MIC (**Figures 1, 2**). Finally, 1/2 MIC of rhubarb water extracts were added to the wells at 0, 24, or 48 h, and then the wells were assayed after incubation for 72 h without shaking. The results showed that biofilm formation was inhibited in a time-dependent manner (**Figure 3**). There was almost no biofilm formation when rhubarb water extracts were added at 0 h, and the results from SEM can be seen in **Figure 1**.

**FIGURE 4:**
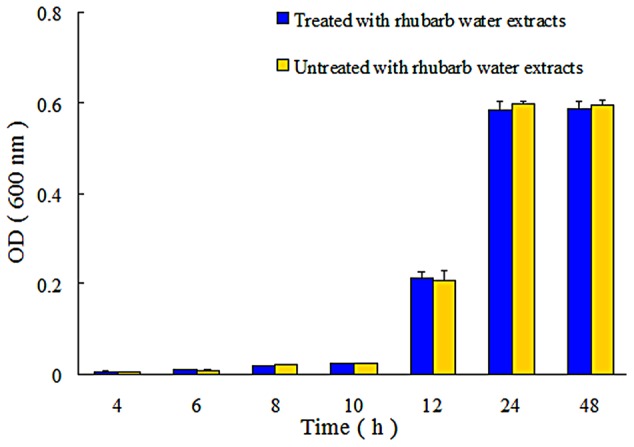
Growth of *S. suis* ATCC700794 in treated and untreated with 1/2 MIC rhubarb water extracts. Data are expressed as means ± standard deviations.

In addition, the extent of growth inhibition of *S. suis* by 1/2 MIC rhubarb extracts was determined. *S. suis* was incubated at 37°C for 48 h, and growth was then monitored at 4, 6, 8, 10, 12, 24, and 48 h by measuring OD_600_. The data indicated that the rhubarb water extracts had no obvious effects on the growth rate of *S. suis* (**Figure 4**).

### Determination of Protein Expression of TCSs, Transcriptional Regulators, and DNA Binding Proteins Using iTRAQ

The expression of TCSs, consisting of HKs and RRs, was measured using an iTRAQ technique (Wang et al., 2011). TCSs were extracted from *S. suis* that was either untreated or treated with 1/2 MIC rhubarb water extracts *in vitro*. HKs were down-regulated in the treated group, especially for G7SK03 and C6GVK0 (**Table 2**). Moreover, most of the RRs were also down-regulated, and B9WXL3 and A4W3Y3 showed a sharp decline (**Table 3**). DNA binding proteins and transcriptional regulators were tested using the iTRAQ technique. Most of the DNA binding proteins were decreased, especially G5KZN4 and G7S463, which sharply declined, in contrast, A4W104 and J7KEW5 showed a significant increase (**Table 4**). There were also some differences for the transcriptional regulators, G5L098 and B9WUV5 were obviously up-regulated, whereas J7KGR5 and G7S5V2 were significantly down-regulated (**Table 5**).

**Table 2:** Changes in histidine kinase proteins expression levels, which were treated with 1/2 MIC rhubarb water extracts.

Accession	Proteins	Fold change
G7SK03^∗^	Signal transduction histidine kinase	0.83
C6GVK0^∗^	Sensor histidine kinase protein	0.85
F4EFQ9	Sensor histidine kinase	0.91
C6GPJ0	Sensor histidine kinase	0.93
A4W106	Signal transduction histidine kinase	0.95

*^∗^*p* < 0.1.*

**Table 3:** Changes in response regulator proteins expression levels, which were treated with 1/2 MIC rhubarb water extracts.

Accession	Proteins	Fold change
B9WXL3^∗∗^	Response regulator receiver protein	0.74
A4W3Y3^∗∗^	Response regulator	0.73
G7S3M8	Response regulator	0.99
G7S4A2	Response regulator	0.99
C6GMK6	Response regulator	1.01
G7SJT4	Response regulator	1.03

*^∗∗^*p* < 0.05.*

**Table 4:** Changes in DNA binding proteins expression levels, which were treated with 1/2 MIC rhubarb water extracts.

Accession	Proteins	Fold change
G5KZN4^∗∗^	DNA polymerase IV	0.40
G7S463^∗∗^	DNA polymerase III	0.73
C6GWQ4	UvrABC system protein A	0.90
G7S6V5	DNA gyrase subunit A	0.92
A4W3D3	Tyrosine recombinase XerD-like	0.96
M1U9Q5	Superfamily I DNA/RNA helicase	0.96
F4ECH1	GTP-sensing transcriptional pleiotropic repressor CodY	0.97
A4W428	DNA polymerase III PolC-type	0.97
M1ULZ5	tRNA N6-adenosine threonylcarbamoyltransferase	0.98
G7SMZ4	Arginine repressor	0.99
G7S183	Chromosome partition protein Smc	1.01
D5AF52	DNA polymerase III	1.03
A4W104^∗∗^	Tyrosine recombinase XerS	1.46
J7KEW5^∗∗^	Uncharacterized protein	1.66

*^∗∗^*p* < 0.05.*

**Table 5:** Changes in transcriptional regulators expression levels, which were treated with 1/2 MIC rhubarb water extracts.

Accession	Proteins	Fold change
G5L098^∗∗^	Transcriptional regulator	1.73
B9WUV5^∗∗^	Transcriptional regulator	1.71
G7S5M4^∗^	Transcriptional regulator	1.30
G7S462	Transcriptional regulator	1.25
G7S4B3	Transcriptional regulator	1.23
F4EEN1	Transcriptional regulator	1.20
G7S711	Transcriptional regulator	1.18
R4NHU3	Transcriptional regulator	1.16
B9WXE7	Transcriptional regulator	1.14
A4W3G1	Transcriptional regulator	1.13
A4VVQ9	Transcriptional regulator	1.10
G7S2W9	Transcriptional regulator	1.09
G7SFA8	Transcriptional regulator	1.07
G7S6T7	Transcriptional regulator	1.07
G7S3G3	Transcriptional regulator	1.06
G7S706	Transcriptional regulator	1.05
M1UW70	Transcriptional regulator	1.03
G7S3P1	Transcriptional regulator	1.03
G7S5A4	Transcriptional regulator	1.03
G7S2P7	Transcriptional regulator	1.02
A4VXD5	Transcriptional regulator	1.01
R4NW63	Transcriptional regulator	1.00
G7S5M2	Transcriptional regulator	1.00
R4NX73	Transcriptional regulator	1.00
G7S1R6	Transcriptional regulator	1.00
G5L1U9	Transcriptional regulator	0.99
G7S354	Transcriptional regulator	0.99
G7S530	Transcriptional regulator	0.94
G7S199	Transcriptional regulator	0.94
G7S3P2	Transcriptional regulator	0.93
G5KZY9	Transcriptional regulator	0.93
G7SCL5	Transcriptional regulator	0.92
G7S2E2	Transcriptional regulator	0.92
G7S387	Transcriptional regulator	0.88
B0M0F2	Transcriptional regulator	0.88
M1UBA1	Transcriptional regulator	0.86
M1UBA1	Transcriptional regulator	0.86
G7SLN9^∗^	Transcriptional regulator	0.83
J7KGR5^∗∗^	Transcriptional regulator	0.82
G7S5V2^∗∗^	Transcriptional regulator	0.81

*^∗^*p* < 0.1, ^∗∗^*p* < 0.05.*

### Determination of Changes in Protein Expression in Different Parts of the Cell

**FIGURE 5:**
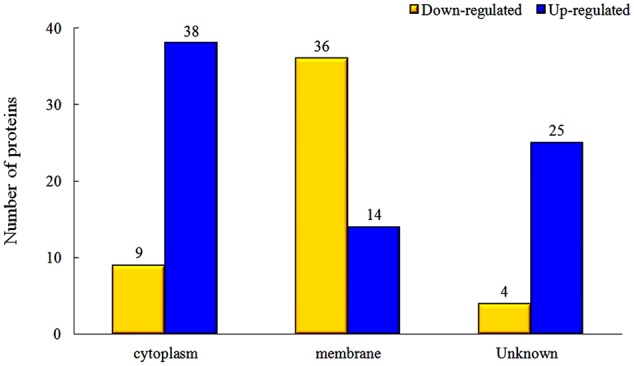
Changes of protein expression levels in different parts of cells, which were treated with 1/2 MIC rhubarb water extracts.

The proteins in different parts of the cell were also investigated in our study, and the results indicated that there were significant changes between rhubarb water extracts-treated and untreated cells. About 36 cellular membrane proteins were down-regulated, and 14 proteins were up-regulated, in treated cells as compared to untreated cells. In contrast, about 38 cytoplasmic proteins were up-regulated and 9 proteins were down-regulated (**Figure 5**).

## Discussion

Rhubarb is a common Chinese herbal medication, and its water extracts are often used in Chinese clinics. Anthraquinones are essential and effective constituent of rhubarb, they can inhibit the growth of viruses, bacteria, and biofilm (Lin et al., 2008; Wierzchacz et al., 2009), so the content of anthraquinones in the rhubarb water extracts were determined. Here, the effect of the rhubarb water extracts on *S. suis* ATCC 700794 biofilm formation inhibition was studied. Both TCP and SEM assays were carry out to test the effects of 1/2 MIC rhubarb extract on inhibition of *S. suis* biofilm formation, and the results demonstrated that the rhubarb water extracts could obviously interrupt *S. suis* biofilms formation (**Figures 1**–**3**); the data from **Figures 2, 3** further indicated that the biofilm formation inhibition rate was dose- and time-dependent. These results were similar to the interruption of *Pseudomonas aeruginosa* biofilm formation by rhein, chrysophanol, and emodin, all of which have been reported to inhibit the bacterial quorum sensing (QS) system and reduce biofilm formation (Ding et al., 2011).

Biofilm formation can be affected by the number of bacteria, and reducing the bacterial number can thus inhibit biofilm formation. In our study, *S. suis* growth inhibition was tested in both untreated and 1/2 MIC rhubarb water extracts-treated cells, and the results indicated that the *S. suis* growth rate was not influenced by the rhubarb water extracts. It appears that inhibition of biofilm formation does not occur through the death of cells.

Transduction system play an important role in biofilm formation (Bhagwat et al., 2001; Parkins et al., 2001; Rasamiravaka et al., 2015; Kim et al., 2016). TCSs can affect autolytic activities, including the synthesis of Atl A, which involved in biofilm formation (Houston et al., 2011; Haghighat et al., 2017). Autolysin can affect bacteria adhesion, which is an essential process in biofilm formation (Heilmann et al., 2003; Biswas et al., 2006; Hussain et al., 2014). Thus the depletion of TCSs can leads to phenotypes directly related to a lack of autolysins, and impairment of both cell aggregation and biofilm formation (Dubrac et al., 2007). In addition, TCSs can receive stimulus signaling [such as autoinducers (AI)] and regulate the QS system, which plays a key role in pathogenic biofilm formation regulation (Rahman et al., 2015; Vasavi et al., 2017). The QS system is a widespread signaling system used by bacteria for cell-to-cell communication. It operates via production, release, and detection of small diffusible signaling molecules (AI), which can promote bacterial communication by interacting with TCSs (Bauer et al., 2015; Ma et al., 2017). Because of their importance in biofilm formation, TCSs have become attractive targets for the development of novel antibacterial, anti-biofilm drugs (Dan et al., 2014). In our study, TCSs were down-regulated expression (**Tables 2, 3**) when sub-MIC rhubarb water extracts were used to treat *S. suis*, and biofilm formation was significantly inhibited (**Figure 2**), this phenomenon agrees with the result from other literature.

**FIGURE 6:**
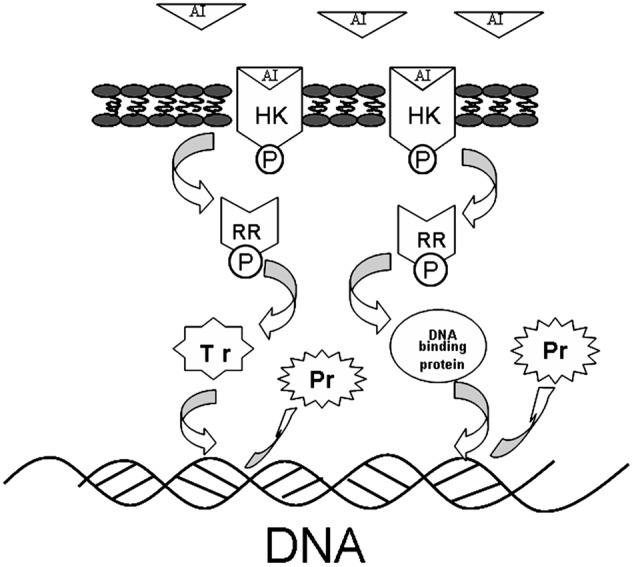
The protein expression influence of TCSs. When the AI is binding or reacting, histidine kinase (HK) will be autophosphorylated, the phosphorus will be transferred to receiver domain of the response regulator (RR), and RR will be dephosphorylated, after interacting with DNA binding proteins or transcriptional regulators. The system will return to its initial state after producing the appropriate cellular responses, which lead to differential protein expression.

The interaction of TCSs with signaling molecules (AI) can regulate transcriptional levels (Mascher et al., 2006; Wu et al., 2014). These functions are controlled by three phosphotransfer reactions: (1) when the signaling molecule is binding or reacting, HK will be autophosphorylated; (2) the actual transfer of the phosphorus group to the receiver domain of the RR (via the activity of the RR); and (3) dephosphorylation of RR, which returns the system back to the initial state (**Figure 6**) (Nobile et al., 2012; Connan and Popoff, 2015). In our study, TCSs were measured by the iTRAQ technique and the levels of most of the HKs and RRs were reduced by treatment with the rhubarb water extracts (**Tables 2, 3**). Compared with the control group, the interaction between TCSs and signaling molecules (AI) could be altered, and message transmission would subsequently be affected. Proteins expression may be different in the rhubarb water extracts-treated group (**Figure 6**). This is consistent with our experimental results as seen in **Figure 5**. There were about 126 altered proteins, 49 protein expression levels were decreased, and 77 protein expression levels were increased (Supplementary Table 1).

Given that TCSs can interact with transcriptional regulators and DNA binding proteins to interrupt transcription (**Figure 6**), in our study, transcriptional regulators and DNA binding proteins were measured using iTRAQ technique. The data can be seen in **Tables 4, 5**, which indicated that both classes of proteins could be affected by the rhubarb extracts. Transcriptional regulators can define a group of coordinated target genes that act together to carry out specific cellular functions (Whiteson et al., 2007; Romero-Rodriguez et al., 2015). They not only regulate primary metabolic processes such as maintenance of fatty acids, amino acid catabolism, and regulation of carbon catabolism, but also affect secondary metabolism (Whiteson et al., 2007). Thus, if transcriptional regulators are perturbed, *S. suis* biofilm formation may be interrupted.

Expression levels of DNA binding proteins were affected in the group treated with the rhubarb water extracts (**Table 4**), especially DNA polymerases III, DNA polymerases IV and tyrosine recombinase XerS. Tyrosine recombinase XerS is a site-specific tyrosine recombinase, which acts by catalyzing the cutting and rejoining of recombining DNA molecules. It is essential for conversion of dimers of bacterial chromosomes into monomers and permitting their segregation at cell division (Ma et al., 2007; Poulter and Butler, 2015). Thus, up-regulating tyrosine recombinase XerS may affect cell division. DNA polymerase III performs coordinated high-fidelity synthesis of leading and lagging strands at the replication fork in a highly progressive manner. It is often induced in response to DNA damage. It plays an important role in DNA damage tolerance by performing translesion DNA synthesis (TLS) (Friedberg, 2005; Nohmi, 2006; Sidorenko et al., 2011). DNA polymerase III is exceptionally fast and accurate, but it is inefficient at synthesizing DNA on damaged templates. When DNA is significantly damaged, the replication fork will collapse and synthesis is difficult to carry out via DNA polymerase III (Robinson et al., 2015). This situation can be addressed by an SOS response, and DNA polymerase IV, as a part of the SOS regulon, can be induced to respond to DNA damage. DNA polymerase IV can lead accurate or error-prone DNA synthesis after replacing DNA polymerase III at the replication fork (Robinson et al., 2015). DNA polymerase IV belongs to Y-family polymerases that are known for their specialized ability to accommodate and bypass lesion-containing DNA (Scotland et al., 2015). They have lower DNA fidelity, are potentially mutagenic, and have a close relationship with bacterial resistance (Jacob and Eckert, 2007). In our study, DNA polymerases III and IV were distinctly down-regulated by the rhubarb water extracts, thus we can speculate that gene repair and bacterial resistance can be affected by the rhubarb extracts, and these results can be further studied in the future.

This work extends knowledge of the pharmacological effects of rhubarb on inhibition of *S. suis* biofilm formation. The inhibitory mechanism was investigated using the iTRAQ technique. The experimental data demonstrated that the *S. suis* biofilm formation could be obviously inhibited by the rhubarb water extracts. Anthraquinones, which are important active ingredient of rhubarb, were measured in the rhubarb water extracts studied here. The reason for the inhibition of biofilm formation was studied in our experiments. Firstly, this inhibition was not achieved by cell killing, which was proven by the experiments on growth inhibition. Secondly, the reduction of biofilm formation may be caused by TCS down-regulation because TCS can affect autolytic activities and QS, which play key role in biofilm formation (Parkins et al., 2001; Rasamiravaka et al., 2015). Thirdly, levels of DNA binding proteins and transcriptional regulators were affected by rhubarb water extracts in the cell. Finally, variations in protein expression levels were measured in different parts of the cell, and about 126 proteins were shown to be affected.

## Author Contributions

Y-HL and ZH guided the experiment. Y-LZ, Y-BY, and QH *Streptococcus suis* experiment. XL, X-YC, HL, and X-YA edited the article. W-YD performed the experiments and wrote the article.

## Conflict of Interest Statement

The authors declare that the research was conducted in the absence of any commercial or financial relationships that could be construed as a potential conflict of interest.
