# Supplementary material for: Sub-Minimum Inhibitory Concentrations of Rhubarb Water Extracts Inhibit Streptococcus suis Biofilm Formation
Source: Front Pharmacol. 2017 Jul 7;8:425. doi: 10.3389/fphar.2017.00425 (PMC5500959; doi:10.3389/fphar.2017.00425)
Supplement: Supplementary file 1 [file Table_1.pdf]

Supplementary Table 1

Table 1. List of significant difference expressed proteins of *Streptococcus suis*, which were treated with 1/2 MIC rhubarb water extracts.

Accession	Proteins	Fold change
G5KZN4	DNA polymerase IV	0.40
G7SIZ9	SIR2 family protein	0.41
G7SHZ3	Bacteriophage protein, putative	0.42
F4EDP5	Putative uncharacterized protein	0.55
G7S3J2	Phosphorylase	0.58
G7S2K0	Phosphotransferase system IIC component, glucose/maltose/N-acetylglucosamine-specific	0.59
G5L351	NsuB	0.60
G7S2J9	Beta-glucosidase/6-phospho-beta-glucosidase/beta-galactosidase	0.63
G7SM56	Ribosomal RNA small subunit methyltransferase H	0.63
G5L158	3-oxoacyl-[acyl-carrier-protein] synthase 2	0.65
G5KZZ1	4-alpha-glucanotransferase	0.66
Q84BJ2	Putative acyltransferase	0.67
G5KZR3	Glutathione S-transferase	0.67
G5L2K3	Putative uncharacterized protein	0.67
G7S2K1	Transcription antiterminator	0.68
G7S229	Preprotein translocase subunit SecG	0.68
E8UKS3	Uncharacterized protein	0.69
G5L259	NADP-dependent glyceraldehyde-3-phosphate dehydrogenase, putative	0.69
M1VJJ3	Glycosyltransferase	0.69
K0FG35	CpsR	0.69
G7S8Q2	Peptidase M22 glycoprotease	0.70
R4NVP5	Peptidyl-prolyl cis-trans isomerase	0.70
M1VK55	Glycosyltransferase	0.70
G7S595	Uridine phosphorylase	0.71
G7S463	DNA polymerase III alpha subunit	0.72
G7S8P5	Phosphoglycerate kinase	0.72
A4W1N1	Putative phosphate ABC transporter	0.72
G7S463	DNA polymerase III	0.73
A4W3Y3	Response regulator	0.73
G7SAL2	3-oxoacyl-[acyl-carrier-protein] synthase 2	0.74
B9WXL3	Response regulator receiver protein	0.74
D5AIH0	Polysaccharide biosynthesis protein	0.75

B9WX95	Asparaginyl-tRNA synthetase	0.75
A4W361	Uridine kinase	0.75
G7S506	Metal-dependent membrane protease	0.76
G7S9H8	Alcohol dehydrogenase	0.76
G7S2K5	Putative uncharacterized protein	0.77
G7S1Y3	ABC-type metal ion transport system, periplasmic component/surface adhesin	0.77
G7SDH9	Putative uncharacterized protein	0.78
D3VNC9	UDP-galactopyranose mutase	0.78
M3T1P2	DegV family protein	0.78
G7S4P5	3-oxoacyl-[acyl-carrier-protein] synthase 2	0.78
G5KX48	ABC-type uncharacterized transport system, permease component	0.78
M1UC12	Fructose-2,6-bisphosphatase	0.78
C6GMK1	Penicillin-binding protein 2b	0.79
C5VWR4	Putative A/G-specific adenine glycosylase	0.79
A4VXV9	Subtilisin-like serine protease	0.79
J7KGR5	Transcriptional regulator	0.82
G7S5V2	Transcriptional regulator	0.81
R4NVJ5	Non-specific DNA-binding protein Dps / Iron-binding ferritin-like antioxidant protein / Ferroxidase	1.38
G7S6K5	Ketosteroid isomerase-like protein	1.38
G7S2N0	Putative uncharacterized protein	1.38
A4W3R6	Ribosome-binding factor A	1.38
A4VYQ2	30S ribosomal protein S17	1.38
A4VS89	Uncharacterized protein conserved in bacteria	1.38
B9WX86	Putative uncharacterized protein	1.39
A4VYP1	30S ribosomal protein S10	1.39
G7SI37	50S ribosomal protein L31 type B	1.40
G7RZ29	Uncharacterized protein	1.40
G7S3A0	Ribosomal silencing factor RsfS	1.40
A4W1B7	Ribosomal protein L10	1.40
G5L1N9	Nucleoid DNA-binding protein	1.40
Q5IRB9	ManO	1.41
A4VSN1	30S ribosomal protein S12	1.42
G5L0K3	Putative uncharacterized protein	1.43
R4NUB1	50S ribosomal protein L18	1.43
D5AIK1	Major cell-binding factor (CBF1)	1.43
R4NJR0	4-oxalocrotonate tautomerase Xylose transport system permease protein xylH	1.43
F4ECY5	Phage protein	1.44
G7S1A4	Phosphotransferase system, mannose/fructose/N-acetylgalactosamine-specif	1.44

	ic component IID	
A4VZF0	50S ribosomal protein L28	1.44
B9WV55	50S ribosomal protein L36	1.45
A4W3Q2	30S ribosomal protein S6	1.45
G5L3A6	Putative uncharacterized protein	1.46
G7SP92	Putative uncharacterized protein	1.46
A4VYQ6	30S ribosomal protein S8	1.46
A4W104	Tyrosine recombinase XerS	1.46
G7SLP3	Negative regulator of copper transport operon	1.47
A4VYQ3	50S ribosomal protein L14	1.47
A4W3W8	30S ribosomal protein S9	1.47
B9WXB0	Endonuclease III	1.47
G7S4L5	Putative uncharacterized protein	1.47
G7S2N4	ABC transporter ATP-binding protein	1.48
G7S5C2	Putative uncharacterized protein	1.50
A4W252	50S ribosomal protein L35	1.50
Q1A7A9	Dpr protein (Fragment)	1.51
A4VZ90	50S ribosomal protein L33	1.51
J7KR12	Keto-hydroxyglutarate-aldolase/keto-deoxy-phosphogluconate aldolase	1.52
A4VYP7	30S ribosomal protein S19	1.52
G7S7E3	Helicase	1.53
A4W3Q0	30S ribosomal protein S18	1.53
M1U9U0	50S ribosomal protein L27	1.55
A4VYQ4	50S ribosomal protein L24	1.56
G7S4E0	Glutamine amidotransferase, class I	1.57
A4VYP8	50S ribosomal protein L22	1.58
B0FYB8	Neprilysin (Fragment)	1.60
G7S197	Putative uncharacterized protein	1.61
R4NW55	Plasmid replication protein Rep and AAA-class ATPase domain protein	1.63
R4NUN9	SSU ribosomal protein S16p	1.63
G7SMM9	Putative uncharacterized protein	1.65
J7KEW5	Uncharacterized protein	1.66
G7SFV1	Putative uncharacterized protein	1.66
R4NTP2	Uncharacterized protein	1.66
C6GNL8	30S ribosomal protein S21	1.67
B9WXF6	Cell wall/surface repeat protein	1.68
B9WUV5	Transcriptional regulator	1.71
R4NL31	MF3-like protein	1.72
G5L098	Transcriptional regulator	1.73
G7S7A9	FAD-dependent pyridine nucleotide-disulfide oxidoreductase	1.74
R4NZ92	TPR repeat-containing protein	1.75

G7SKQ9	Phosphoribosylformylglycinamidine synthase domain-containing protein	1.75
M1VRG0	Uncharacterized protein	1.76
G7S568	Putative uncharacterized protein	1.81
B9WSL5	Sua5/YciO/YrdC/YwlC family protein	1.83
E8UNR8	30S ribosomal protein S20	1.85
G5KZ73	NADH:flavin oxidoreductase / NADH oxidase family protein	1.87
G5KZ86	Phosphatidylserine/phosphatidylglycerophosphate/ cardiolipin synthase-like protein	2.00
M1UAF6	Glutaredoxin-like protein	2.00
G5KY37	Putative uncharacterized protein	2.10
G7SM99	Type I site-specific restriction-modification system, R (Restriction) subunit and related helicase	2.11
E9NQ13	CPS16F	2.22
A4W2G9	Cation transport ATPase	2.59
G7SD52	ABC superfamily ATP binding cassette transporter, membrane protein	2.63
C6GT52	Chloramphenicol acetyltransferase	3.38
G7S265	Copper chaperone	3.69
G5L0Y1	ABC-type transport system involved in Fe-S cluster assembly, permease component	4.27
